# Th1/Th17 Cell Induction and Corresponding Reduction in ATP Consumption following Vaccination with the Novel *Mycobacterium tuberculosis* Vaccine MVA85A

**DOI:** 10.1371/journal.pone.0023463

**Published:** 2011-08-26

**Authors:** Kristin L. Griffiths, Ansar A. Pathan, Angela M. Minassian, Clare R. Sander, Natalie E. R. Beveridge, Adrian V. S. Hill, Helen A. Fletcher, Helen McShane

**Affiliations:** The Jenner Institute, Oxford University, Oxford, United Kingdom; Institut Pasteur, France

## Abstract

Vaccination with Bacille Calmette-Guérin (BCG) has traditionally been used for protection against disease caused by the bacterium *Mycobacterium tuberculosis* (*M.tb*). The efficacy of BCG, especially against pulmonary tuberculosis (TB) is variable. The best protection is conferred in temperate climates and there is close to zero protection in many tropical areas with a high prevalence of both tuberculous and non-tuberculous mycobacterial species. Although interferon (IFN)-γ is known to be important in protection against TB disease, data is emerging on a possible role for interleukin (IL)-17 as a key cytokine in both murine and bovine TB vaccine studies, as well as in humans. Modified Vaccinia virus Ankara expressing Antigen 85A (MVA85A) is a novel TB vaccine designed to enhance responses induced by BCG. Antigen-specific IFN-γ production has already been shown to peak one week post-MVA85A vaccination, and an inverse relationship between IL-17-producing cells and regulatory T cells expressing the ectonucleosidease CD39, which metabolises pro-inflammatory extracellular ATP has previously been described. This paper explores this relationship and finds that consumption of extracellular ATP by peripheral blood mononuclear cells from MVA85A-vaccinated subjects drops two weeks post-vaccination, corresponding to a drop in the percentage of a regulatory T cell subset expressing the ectonucleosidase CD39. Also at this time point, we report a peak in co-production of IL-17 and IFN-γ by CD4^+^ T cells. These results suggest a relationship between extracellular ATP and effector responses and unveil a possible pathway that could be targeted during vaccine design.

## Introduction

*Mycobacterium tuberculosis* (*M.tb*), the causative agent of tuberculosis (TB), infects ∼30% of the World's population and is endemic in Asia and sub-Saharan Africa. *M.tb* is transmitted by aerosol and infects macrophages in the lung. Infection with *M.tb* can result in acute (primary) disease, or, more commonly, remain latent. In 10% of cases, this latent infection reactivates later in life causing disease, which is usually pulmonary but can occur in other organs including spleen, stomach, bowel or brain. Co-infection with HIV, also endemic in TB-endemic areas, results in a significant increase in the risk of reactivation of this latent infection [1], [2], [3]. Bacille Calmette-Guérin (BCG), the current TB vaccine, is an attenuated form of *M.bovis* and offers varying degrees of protection. Notably, the level of protection is lowest in areas endemic for *M.tb* and the development of a novel, more effective vaccine against TB is urgently needed.

Modified Vaccinia virus Ankara expressing antigen 85A (MVA85A), a secreted and highly immunogenic protein common to both BCG and *M.tb*, is a vaccine designed to enhance the low level of T cell responses induced by BCG through expansion of antigen 85A-specific T cells [4], [5]. The MVA85A-induced interferon (IFN)-γ response has been well-characterised across several groups of vaccinated individuals and peaks 1–2 weeks post-vaccination [4], [5], [6], [7], [8]. Recently, however, other immunological parameters have been investigated, in particular those pertaining to immune regulation. MVA85A has been shown to induce a reduction in transforming growth factor (TGF)-β in the serum [9] as well as a reduction in percentages of CD25^+^Foxp3^+^CD39^+^ Treg in peripheral blood monocytes (PBMC) from vaccinated subjects [10]. CD39, an ectonucleosidase triphosphate diphosphohydrolase (eNDTPase; apyrase) hydrolysing extracellular adenosine triphosphate (ATP) to adenosine monophosphate (AMP) [11], is expressed on leukocytes, including neutrophils and T cells. In humans, CD4^+^ T cells can be divided according to their expression of CD25, CD39 and Foxp3, with CD4^+^CD25^+^CD39^+^Foxp3^+^ cells representing a regulatory population [12]. ATP is released into the extracellular environment as a natural process during inflammation [13] as well as being released by dead cells. It has been identified as a proinflammatory agent and is known to activate the NALP3 inflammasome through binding to the P2X7 receptor and inducing a K^+^ efflux, as well as activating the Pannexin-1 channel [14], [15]. The combination of these events drives the cleavage of pro-IL-1, induced by Toll-like receptor (TLR) activation, to IL-1β by Caspase-1 [16]. IL-1β, in synergy with IL-6, has in turn been shown to induce IL-17 production by Th17 cells [17]. Reduced percentages of Foxp3^+^CD39^+^ Treg have been described in PBMC of patients with multiple sclerosis compared to healthy donors [18], [19], and the CD39^+^ cells that were present had impaired ATP-hydrolysing capacity [19], providing evidence for a link between CD39 expression on Treg cells and a function in regulating inflammation through controlling extracellular ATP levels.

Here we show that CD39^+^ Treg percentages drop 2 weeks post-MVA85A vaccination, coincident with a drop in ATP consumption by PBMC from MVA85A-vaccinated subjects. This also coincides with an increase in percentages IFN-γ and IL-17 double-producing CD4^+^ T cells.

Clearance and control of *M.tb* infection is at least partly dependent upon interferon (IFN)-γ production by CD4^+^ T helper 1 (Th1) cells [20], however IL-17 has recently been identified as being induced by *M.tb* in murine lungs following vaccination with adjuvanted peptides derived from ESAT-6, an immunodominant secreted protein specific to *M.tb* [21]. Vaccination of mice lacking IL-23 subunits, the cytokine essential for Th17 expansion, resulted in the loss of accelerated vaccine-induced recruitment of Th1 cells to the lungs following *M.tb* infection, suggesting that IL-17-producing cells (Th17) contribute to vaccine-induced protection against *M.tb* challenge through recruitment of Th1 cells to the lung. These hypotheses are supported by a different study in which mice were vaccinated with either BCG or BCG followed by a construct designed to produce anti-IL-12 antibodies within the animal, or with the anti-IL-12-inducing construct alone [22]. Following an *M.tb* challenge, results showed higher bacterial load (cfu) in lungs and spleen from mice with anti-IL-12 antibodies compared to no treatment, but no difference in cfu between BCG or BCG+anti-IL-12 groups, which both had significantly lower cfu than unvaccinated mice. Interestingly, higher IL-17 and IL-6 levels were detected in the vaccinated compared to the unvaccinated groups, suggesting that control during primary intravenous infection depends on a Th1 response, but on an IL-17-driven response following vaccination.

Further support for the involvement of IL-17 in control of *M.tb* infection comes from a recent study comparing cytokine levels in tuberculin skin test (TST) negative and TST positive (considered latently infected) individuals in a TB endemic area. These results showed that IL-17, IL-23 and RORγt, the transcription factor implicated in Th17 development, were downregulated in TST^+^ individuals [23] suggesting that higher IL-17 production favours clearance or control of *M.tb*.

MVA85A has previously been shown to increase interleukin (IL)-17 production in both humans and cattle [10], [24]. Furthermore, in cattle, vaccine-induced IL-17 production both pre- and post- *M.bovis* challenge has been correlated with vaccine-induced protection against TB disease [24]. IL-17 has also been detected in whole blood of MVA85A-vaccinated adolescents and children, where the IL-17^+^ cells were also found to produce IFN-γ, tumour necrosis factor (TNF)-α and IL-2 [25]. Here we suggest a possible link between CD39^+^ Treg cells and potentially protective MVA85A-induced IFN-γ and IL-17 production.

## Results

### ATP consumption following MVA85A vaccination follows a distinct pattern and can be inhibited using an apyrase inhibitor

**Figure 1:**
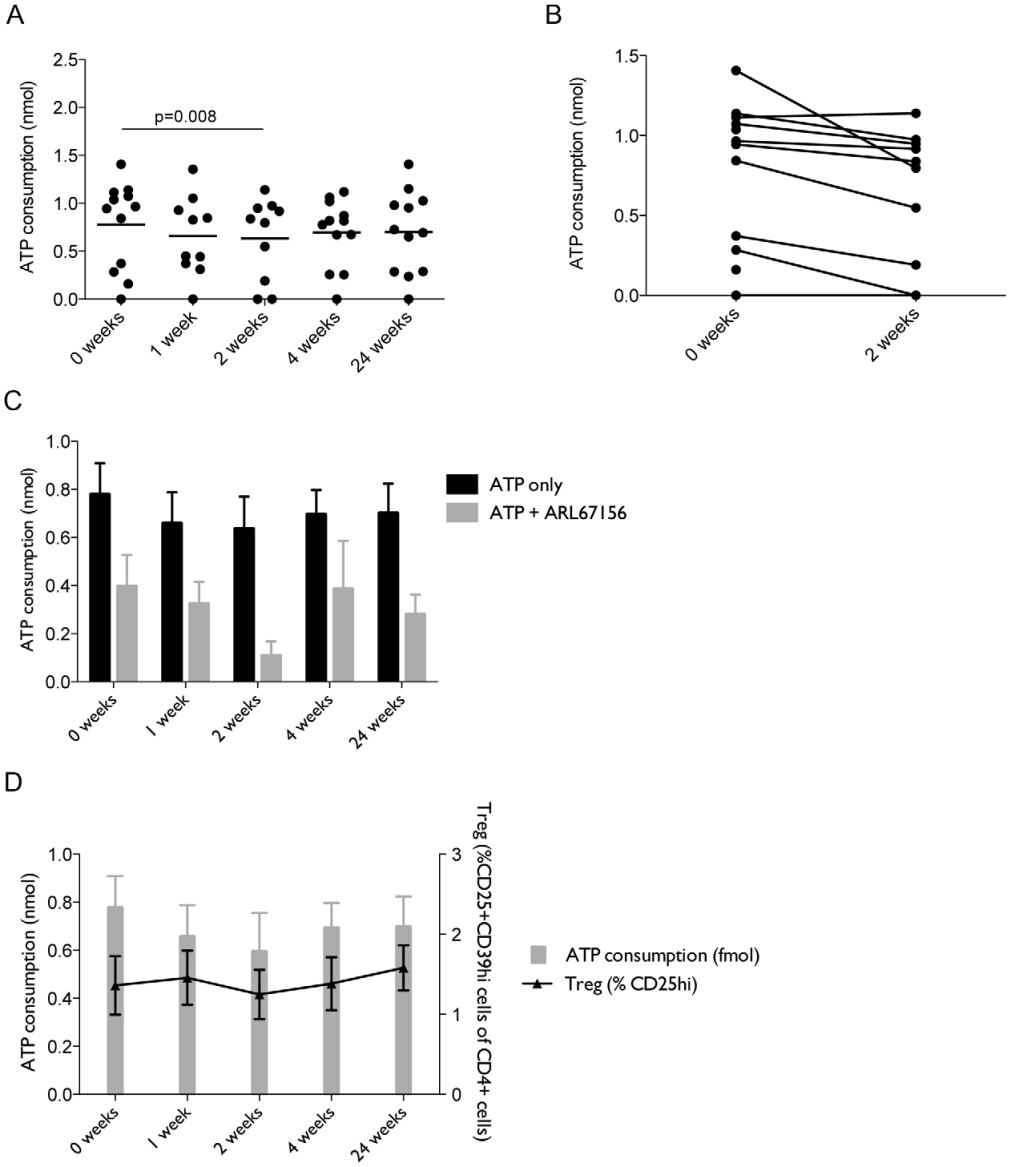
ATP consumption by PBMC and CD39^+^ Treg percentages dip 2 weeks post-vaccination. PBMC from MVA85A-vaccinated subjects were plated out at 5×10^4^ cells/well in 50 µL. Cells were incubated with either 50 µM ATP or 50 µM+100 µM ARL67156 before addition of the luciferase reagent. A standard curve starting at 50 µM ATP was set up and negative controls were cells with no ATP added. (A) Shows change in ATP consumption over time post-vaccination. (B) Paired representation of change in ATP consumption between 0 and 2 weeks post-MVA85A. Effect of addition of ARL67156 is show in (C). (n = 10–12). Note that the observation of a greater effect of the inhibitor is potentially due to saturation of binding sites for ATP by the inhibitor at this timepoint, whereas the greater percentage of CD39^+^ cells present at other timepoints meant the concentration of ARL67156 was not high enough to completely block all available binding sites. Percentages of CD25^+^CD39^+^ Treg in MVA85A-vaccinated subjects were calculated as a percentage of CD4^+^ T cells and shown in (D), plotted over ATP consumption.

**Figure 2:**
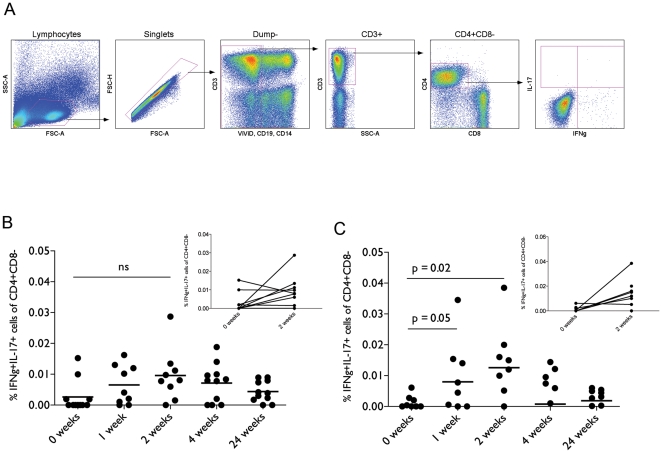
IL-17 and IFN-γ production in PBMC peaks 2 weeks post-vaccination. PBMC from vaccinated subjects were stimulated with Ag85A peptide pools with or without 100 uM ARL67156. No stimulation and phorbol 12-myristate 13-acetate with ionomycin were used as negative and positive controls. Percentages shown are unstimulated subtracted from Ag85A stimulation. Following staining, cells were gated as shown in (A): Lymphocytes were gated for on FSC vs. SSC. Singlets were then gated and dead cells, B cells and monocytes were gated out. CD3^+^ cells were selected for CD4^+^CD8^−^ cells. Antigen-specific cytokine expression from these cells was evaluated. Cells expressing both IL-17 and IFN-γ were quantified and shown in (B). The effect of ARL67156 on cytokine expression was investigated by addition during ICS stimulation (C).

Consumption of extracellular ATP was measured in PBMC from vaccinated subjects at 0, 1, 2, 4 and 24 weeks post-vaccination using the CellTiter-Glo cell viability assay and plotting against a standard curve. There was a significant difference in ATP consumption 2 weeks post-vaccination compared to baseline (p = 0.008) (Fig. 1A). Paired analysis between 0 and 2 weeks is shown in figure 2B. In order to verify that ATP consumption was attributable to the action of an apyrase, cells were treated with ARL67156 at the time of ATP addition, which reduced ATP consumption (Fig. 1C).

Regulatory T cells, defined here as CD4^+^CD25^+^CD39^hi^, have previously been shown to decrease in number post-vaccination with MVA85A [10]. Since CD39 is an eNDTPase metabolising ATP, percentages of these cells in PBMC of vaccinated subjects were compared to levels of ATP consumption. There was a small dip in percentages of CD39^+^ Treg cells between 1 and 2 weeks post-vaccination and followed the pattern of ATP consumption (Fig. 1D).

### IFN-γ and IL-17 double positive cells peak 2 weeks post-vaccination

In PBMC from healthy MVA85A-vaccinated subjects, T cells producing only IL-17 were not detectable by intracellular cytokine staining, using a peptide pool of 66 Ag85A peptides as the stimulant (data not shown). In contrast, CD4^+^ T cells producing both IFN-γ and IL-17 simultaneously in response to stimulation with Ag85A peptides were readily detected in response to the same stimulant. IFN-γ^+^IL-17^+^ cells (gating shown in Fig. 2A) peaked 2 weeks post-vaccination (Fig. 2B). Since there appeared to be CD39 activity in the PBMC samples (Fig. 1C), the effect of ARL67156 treatment on antigen-specific cytokine production was examined. Addition of ARL67156 enhanced production of both cytokines both 1 and 2 weeks post-vaccination and the change in cytokine production compared to baseline in this experiment was significant (p = 0.047 at 1 week and 0.02 at 2 weeks post-vaccination) (Fig. 2C).

### IL-17^+^ and IFN-γ^+^IL-17^+^ cells are present at a higher frequency in whole blood compared to PBMC of vaccinated subjects

**Figure 3:**
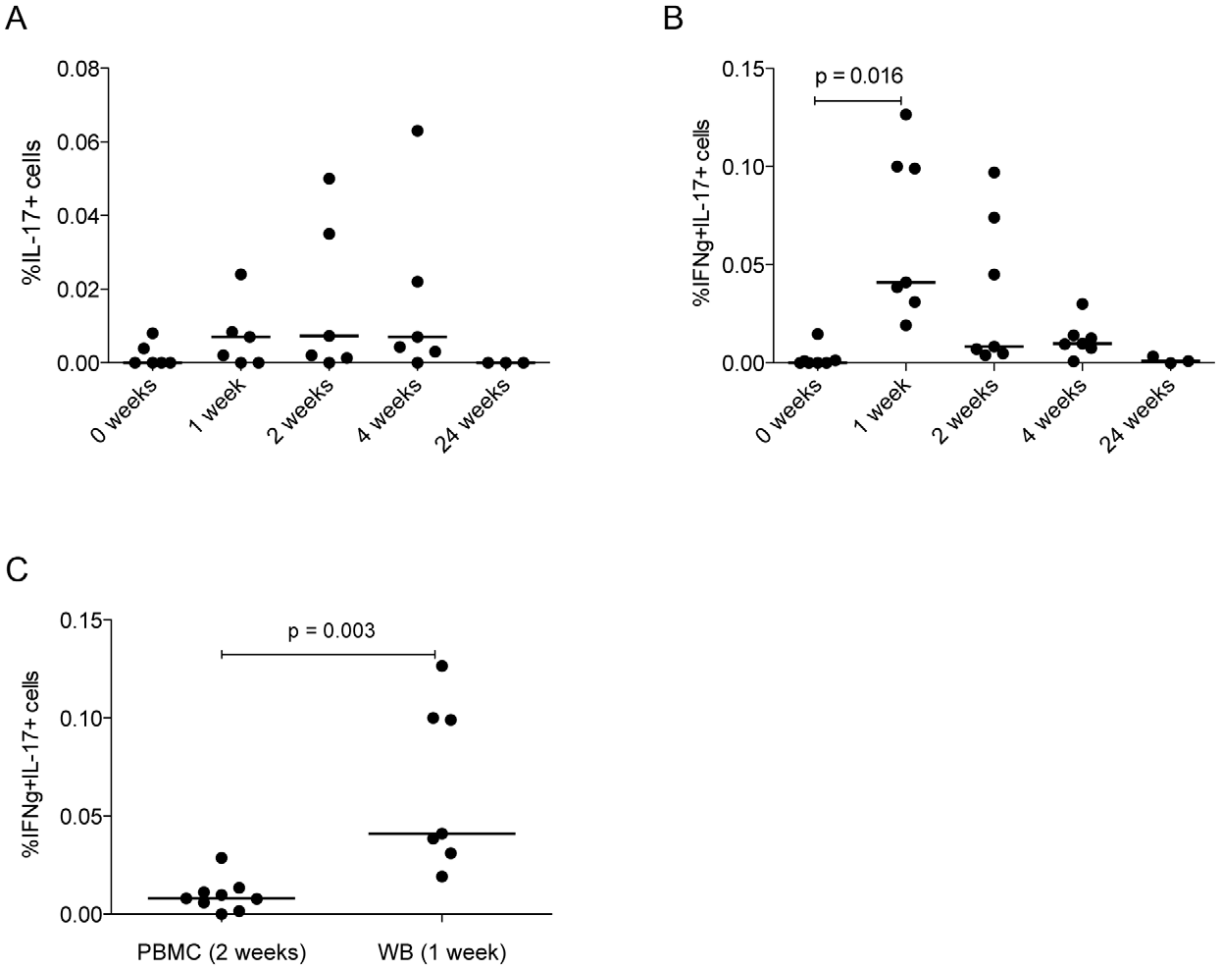
IL-17 and IFN-γ production in whole blood peaks 1 week post-vaccination. Whole blood (WB) from vaccinated subjects was stimulated for 6 h with a pool of 66 Ag85A peptides. Phytohaemagglutinin (PHA)-treated and untreated cells were used as positive and negative controls respectively. Lymphocytes were gated for and cytokine-expressing cells quantified as described above. Percentages of IL-17^+^ and IFN-γ^+^IL-17^+^ cells responding to the Ag85A peptide pool (with the percentages from unstimulated cells subtracted) are shown in (A) and (B). Differences in percentages of cells at the relevant peak time point in PBMC vs. WB is shown in (C). n = 7.

Other trials involving vaccination with MVA85A have investigated cytokine responses in whole blood as opposed to PBMC. One marked difference between these two compartments is that IFN-γ-producing CD8^+^ T cells are readily detectable in whole blood but not in PBMC following vaccination ([5], [26] and Satti, I. unpublished data). To directly compare CD4^+^ T cell cytokine production in these two compartments, we investigated IL-17 and IFN-γ production following stimulation with Ag85A peptides in whole blood and PBMC from the same BCG-vaccinated healthy subjects. Whole blood was stimulated with peptide pools of 85A. Both IL-17^+^ and IFN-γ^+^IL-17^+^ cells peaked 1 week post-vaccination (Figs. 3A & B), in contrast to the undetectable IL-17 response and the peak of IFN-γ^+^IL-17^+^ cells observed 2 weeks post-vaccination in PBMC. The magnitude of response was also significantly higher one week post-vaccination in whole blood, with a mean of 0.0548% (±0.0355) compared to 0.0065% (±0.0063) for PBMC, p = 0.002 following stimulation with the Ag85A peptide pool. Furthermore, there was a significant difference in peak response comparing whole blood and PBMC following Ag85A peptide pool stimulation (Fig. 3C) (mean ± SD for WB (1 week post-vaccination): 0.0548% (±0.0355); PBMC (2 weeks post-vaccination): 0.0096% (±0.0083), p<0.001).

## Discussion

*M.tb* is a resilient intracellular pathogen that has evolved to survive successfully inside human macrophages in a delicate balance with the host's immune system. Should this balance be tipped in favour of the pathogen, the result is potentially fatal active disease. Vaccination is therefore essential in order to either prevent initial infection or, failing that, to prevent development of active TB disease. Consequently, it is important to understand the types of immune cells that should optimally be induced by vaccination and the mechanisms through which these cells can be induced. As well as measuring effector responses in terms of Th1 immunity, it is also important to measure other types of effector responses as well as the corresponding regulatory response induced by vaccination as protection induced by any vaccine will be the outcome of the balance of all these responses.

Here we show that there is a relationship between ATP consumption, and IFN-γ and IL-17 expression by CD4^+^ T cells following vaccination with MVA85A, which also relates to percentages of CD39^+^ Treg in the PBMC. In healthy BCG and MVA85A-vaccinated subjects, CD39^+^ Treg numbers decrease post-vaccination, with a maximum decrease 2 weeks post-vaccination. This is associated with a decrease in ATP consumption, suggesting that ATP consumption is driven at least in part by CD39^+^ Treg. Strikingly, at 2 weeks post-vaccination, we also show an increase in the percentage of IFN-γ^+^IL-17^+^ cells. IFN-γ^+^IL-17^+^ cells have been previously been described in autoimmune diseases [19] and ulcerative colitis [27] as well as in response to mycobacterial antigens [28]. Our data support the hypothesis that extracellular ATP may help to drive the development of these cells. This hypothesis is supported by the fact that IL-17^+^ and IFN-γ^+^IL-17^+^ cells are more readily detectable in whole blood compared to PBMC; the extracellular environment of whole blood is far more complex than that of PBMC and our experiments have shown a higher concentration of ATP in whole blood compared to PBMC following stimulation. Furthermore, ATP has previously been shown to induce IL-17 production by T cells in mice [29].

The link between these observations following MVA85A vaccination is still under investigation. One mechanism that could contribute to increased cytokine production is activation of the NALP3 inflammasome. This leads to IL-1β production, which in turn acts on induction of CD4^+^ T cells to produce IL-17 [17]. Since CD39 metabolises ATP, a vaccine-induced reduction in circulating CD39^+^ Treg may result in increased concentrations of pro-inflammatory extracellular ATP, known to act through the P2X7 receptor to activate the inflammasome [14], [30]. Adding to this effect, a decrease in circulating CD39^+^ Treg would also reduce the concentration of breakdown products of ATP, such as adenosine, which is known to act in an inhibitory fashion through the A2A receptor on activated T cells [31].

A further contributing factor to inflammasome activation and IL-17 induction might be the viral vector MVA, which has been found to activate the NALP3 inflammasome in THP-1 cells following its endocytosis [32].

The role of IFN-γ^+^IL-17^+^ cells in protection against mycobacterial infection is not clear, however both cytokines individually are known to be important in vaccine-induced protection. So far in mice, it has been shown that ablation of an IL-17 response following both vaccination (antigen-specific production) [21] and high-dose *M.tb* challenge (production by innate cells) [33] leads to reduced protection against *M.tb* infection. However, it would also appear that pathology induced as a result of prolonged exposure to mycobacterial antigens is IL-17-dependent [34]. Measurement of IL-17 production in lungs following either vaccination or *M.tb* infection is not possible in human studies so it is difficult to draw comparisons and to predict the role of IL-17, especially since the mouse model of *M.tb* disease is not an ideal representation of human disease. As discussed above, it has been found that IL-17 levels in blood in both humans and cattle correlate with protection against mycobacterial exposure [23] and mycobacterial infection [24] respectively, so it may be, as proposed by Torrado and Cooper [35], that while IL-17 may be essential in vaccine-induced control of TB disease, it needs to be under tight regulation by other aspects of the immune response in order to avoid induction of immunopathology.

These findings demonstrate vaccine-mediated induction of a subset of IFN-γ^+^IL-17^+^ cells, whose peak corresponds with a reduction in the ability of these cells to hydrolyse ATP in a CD39-mediated manner. Should this cell subset prove to be protective in vaccine-induce protection against TB disease, this pathway represents a potential target for manipulation for their enhancement.

Knowledge of mechanisms through which IL-17-, and IFN-γ and IL-17-producing cells can be induced is important with regard to vaccine development, with the next step being to determine their role in vaccine-induced protection or pathology.

Furthermore, it is important to investigate regulatory aspects of cellular immunity, as the total outcome of any vaccine-induced immunity will be the result of a balance between both effector and regulatory responses. The work here helps to dissect this interaction between regulatory and effector responses following vaccination and provides potential avenues for manipulation of immune responses in order to provide improved vaccine-induced protection.

## Materials and Methods

### Vaccine study participants

PBMC were from subjects recruited for a trial approved by the review committees indicated below. Subjects (aged 18–50) were recruited on the basis of prior BCG vaccination (maximum Mantoux test 15 mm, <10 sfc/million ELISpot counts in response to ESAT-6 and CFP10 peptide pools) and were seronegative for HIV and hepatitis B and C viruses. The trial was registered on the clinical trials database (ClinicalTrials.gov ID: NCT00465465). Subjects from whom samples were taken received a dose of MVA85A at 1×10^8^ pfu as two intradermal injections, administered simultaneously, one in each arm. The MVA85A vaccine was manufactured to Good Manufacturing Practice (GMP) by Impfstoffwerk Dessau-Tornau (IDT) Biologika GmbH in Germany. Samples from weeks 0, 1, 2, 4 and 24 were investigated.

The efficacy and safety data from this trial is in the process of being written up but has yet to be published; immunological data from the same trial has been published in Beveridge et al. 2008 [26].

### Ethics statement

All clincal trials are fully approved by the ethical and regulatory agencies (Centre of Research: Ethical Campaign, and Medicines and Healthcare Products Regulatory Agency), and also local GMO and NHS committees as required (the Gene Therapy Advisory Committee), and full written consent was obtained from each subject prior to enrolment in the trial. Storage of samples for exploratory immunological analyses is fully ethically approved.

### PBMC preparation

PBMC from vaccinated subjects were cryopreserved in liquid nitrogen at time of acquisition in aliquots of 5×10^6^ cells in 50% fetal bovine serum (FBS; Biosera Ltd.), 40% RPMI 1640, 10% dimethylsulphoxide (DMSO; both from Sigma Aldrich). Prior to use, cells were thawed in 9 mL R10 (10% FBS, 2 mM L-glutamine, 100 U/mL penicillin, 100 ug/mL streptomycin in RPMI 1640). Cells were treated with 67.2 U/mL Benzonase (Novagen) for at least 2 hours at 37°C in 5% CO_2_. Cells were washed and resuspended to ∼1×10^6^ cells/mL and counted using a CASY cell counter (Schärfe System, GmbH).

### Antibodies and reagents

Anti-human antibodies (Pacific blue anti-CD19, eFluor450 anti-CD19, Pacific blue anti-CD14, eFluor450 anti-CD14, PE-Cy5 anti-CD3, APC-AlexaFluor780 anti-CD8, APC anti-CD25, PE-Cy7 anti-CD39, FITC anti-IFN-γ, PE anti-IL-17) were obtained from eBioscience, and Qdot655 anti-CD4 and the ViViD Live/Dead cell stain were from Invitrogen. The Cytofix/Cytoperm intracellular staining kit was from BD Biosciences. ATP was purchased from Millipore, and ARL67156 from Tocris Bioscience. The CellTiter-Glo cell viability kit was from Promega. Brefeldin A was supplied by Sigma Aldrich and GolgiStop by BD.

### Intracellular cytokine staining

Cells were resuspended at 1×10^6^ cells/mL and each sample divided into seven 1 mL aliquots in 5 mL polystyrene round bottom tubes (BD Falcon). Cells were stimulated for 18 hours at 37°C in 5% CO_2_ with one of the following: no stimulation, no stimulation +100 uM ATP, no stimulation +100 uM ARL67156, 0.2 ug/mL phorbol 12-myristate 13-acetate (PMA) and 2 ug/mL ionomycin, 2 ug/mL Ag85A peptide pool, 2 ug/mL Ag85A peptide pool +100 uM ATP, 2 ug/mL Ag85A peptide pool +100 uM ARL67156. After 2 hours, 5 ug/mL Brefeldin A and 0.7 uL GolgiStop (BD Biosciences) were added.

After stimulation, cells were sedimented at 1300 rpm for 5 min at 4°C and transferred to a flexible 96 well plate for staining.

Cells were stained with ViViD Live/Dead cell stain prior to surface staining with eFluor450 anti-CD19, eFluor450 anti-CD14, APC anti-CD25 and PE-Cy7 anti-CD39. Cytofix/Cytoperm was used to permeabilise cells prior to staining intracellularly with PE-Cy5 anti-CD3, Qdot655 anti-CD4, APC-AlexaFluor780 anti-CD8, FITC anti-IFN-γ and PE anti-IL-17. Following permeabilisation, washes between stains included sedimentation for 5 min at 1800 rpm, 4°C.

Staining was analysed using an LSR II flow cytometer (BD Biosciences).

### Whole blood ICS

Freshly collected heparinised whole blood from MVA85A-vaccinated volunteers was stimulated for 10–11 hours at 37°C with either recombinant Ag85A, a pool of 66 peptides of Ag85A, PPD or BCG. No stimulation and Phytohaemagglutinin were used as negative and positive controls, respectively. After 5–6 hours, Brefeldin A was added for the final 5 hours. Cells were harvested by adding 1 mL FACS Lysing Solution (BD Bioscience) and sedimenting. Cells were frozen in 10% DMSO in FCS and stored at −80°C.

### ATP consumption assay

ATP consumption was measured as described Borsellino *et al.* [18]. Briefly, PBMC from subjects were resuspended at 1×10^6^ cells/mL and aliquoted into 6 wells/sample of a white 96 well plate (Nunc) at 5×10^4^ cells/well. Cells were treated with either nothing, 50 uM ATP or 50 uM ATP +100 uM ARL67156 for 10 min at room temperature. One volume CellTiter-Glo solution was added to each well and cells were incubated for a further 10 min at room temperature in the dark. Luminescence was recorded using a Varioskan Flash spectral scanning multimode reader (Thermo Scientific). ATP consumption was calculated using a standard curve of known ATP concentrations and expressed in nmol.

### Statistical analysis

Given the non-parametric nature of the data, Wilcoxon Sign Rank Tests and Mann-Whitney Tests were performed as appropriate tests for statistical analysis.
